# Conjugate vaccine serotypes persist as major causes of non-invasive pneumococcal pneumonia in Portugal despite declines in serotypes 3 and 19A (2012-2015)

**DOI:** 10.1371/journal.pone.0206912

**Published:** 2018-11-02

**Authors:** Andreia N. Horácio, Catarina Silva-Costa, Elísia Lopes, Mário Ramirez, José Melo-Cristino, on behalf of the Portuguese Group for the Study of Streptococcal Infections

**Affiliations:** Instituto de Microbiologia, Instituto de Medicina Molecular, Faculdade de Medicina, Universidade de Lisboa, Lisboa, Portugal; Universidad Nacional de la Plata, ARGENTINA

## Abstract

Non-invasive pneumococcal pneumonia (NIPP) is a frequent cause of morbidity and mortality worldwide. The 13-valent pneumococcal conjugate vaccine (PCV13) was included in the national immunization program of children living in Portugal in 2015. Until then, PCV7 (since late 2001) and PCV13 (since early 2010) were given through the private market. We determined the serotype distribution and antimicrobial susceptibility of isolates causing adult NIPP in 2012–2015 and compared the results with previously published data (2007–2011). There were 50 serotypes among the 1435 isolates. The most common were serotypes: 3 (14%), 11A (8%), 19F (6%), 23A (5%), 6C (5%), 19A (4%), 23B (4%), 9N (4%) and non-typable isolates (4%). When considering data since the availability of PCV13 for children in the private market, the proportion of PCV13 serotypes declined from 44.0% in 2010 to 29.7% in 2015 (p < 0.001), mainly due to early decreases in the proportions of serotypes 3 and 19A. In contrast, during the same period, PCV7 serotypes (11.9% in 2012–2015) and the serotypes exclusive of the 23-valent polysaccharide vaccine (26.0% in 2012–2015), remained relatively stable, while non-vaccine types increased from 27.0% in 2010 to 41.9% in 2015 (p<0.001). According to the Clinical and Laboratory Standards Institute (CLSI) breakpoints, penicillin non-susceptible and erythromycin resistant isolates accounted for 1% and 21.7%, respectively, of the isolates recovered in 2012–2015, with no significant changes seen since 2007. Comparison of NIPP serotypes with contemporary invasive disease serotypes identified associations of 19 serotypes with either disease presentation. The introduction of PCV13 in the national immunization program for children from 2015 onwards may lead to reductions in the proportion of NIPP due to vaccine serotypes but continued NIPP surveillance is essential due to a different serotype distribution from invasive disease.

## Introduction

Pneumococcal pneumonia is among the most frequent causes of death due to infection worldwide, particularly among young children and older adults [1]. Non-invasive pneumococcal pneumonia (NIPP) is three to ten times more frequent than bacteremic pneumonia [2], but studies evaluating NIPP are less abundant than those evaluating invasive pneumococcal disease (IPD).

After the introduction of pneumococcal conjugate vaccines (PCVs) for children, several studies reported a reduction of IPD in children [3,4]. Given that young children are the main reservoirs and transmitters of pneumococcus in the community and because the PCVs reduce pneumococcal colonization, several studies also reported reductions of IPD due to vaccine serotypes in the non-vaccinated population [5–8].

Despite the lower number of studies, there is also evidence of herd protection in adult NIPP [9–12]. One study from the Netherlands suggested that, based on the similarity of vaccine serotype trends between NIPP and IPD, their national IPD data could be used to extrapolate the trends of NIPP [13]. However, there are also reasons to question predictions of NIPP trends from IPD data in all settings. Perhaps the most significant is that serotype distribution and the proportion of disease that is due to vaccine serotypes differs geographically and between IPD and NIPP [14,15]. Moreover, vaccine serotypes are free to circulate in unvaccinated people so that, especially in countries where the PCVs are not included in national immunization programs, these can persist as causes of disease, both of NIPP and IPD.

In Portugal, PCVs were available only outside the national immunization program for pediatric use until mid-2015. The first PCV to become available was the 7-valent formulation (PCV7), in late-2001. Although the cost of vaccination was fully supported by the parents, the initially modest uptake of PCV7 increased steadily, reaching 75% in 2008 [16]. A 13-valent formulation (PCV13) replaced PCV7 in early-2010 but uptake declined, although it stayed above 60% [17]. In June 2015, PCV13 was included in the national immunization program to be given free of charge to all children born from January 2015 onwards, with a 2+1 schedule [17]. Besides children, sequential vaccination with PCV13 and the 23-valent pneumococcal polysaccharide vaccine (PPV23) is recommended since 2015 by the national health authorities, but only for specific risk groups [18]. In addition, two Portuguese medical societies (respiratory society and general practitioner society) have issued recommendations for the sequential vaccination with PCV13 and PPV23 of all immunocompetent adults ≥65 years [19,20]. Still, pneumococcal vaccine uptake in adults is generally believed to be low, with a study finding that <9% of all adults ≥65 years had received PPV23 [21,22].

In a previous study we analyzed the distribution of serotypes in a randomly selected sample of 100 isolates/year collected from adult NIPP between 1999 and 2011 [14]. In the present study we aimed to gain further insights regarding vaccine serotype trends in adult NIPP in the years that followed. We characterized isolates causing adult NIPP throughout Portugal from 2012 to 2015 for serotype distribution and antimicrobial susceptibility. We also wanted to compare the NIPP data with contemporary adult IPD data obtained by the same network.

## Materials and methods

### Ethics statement

The study was approved by the Institutional Review Board of the Centro Académico de Medicina de Lisboa. These were considered surveillance activities and were exempt from informed consent. All methods were performed in accordance with the relevant guidelines and regulations. The data and isolates were de-identified so that these were irretrievably unlinked to an identifiable person.

### Bacterial isolates

Isolates were provided by a laboratory-based surveillance system that includes 30 microbiology laboratories throughout Portugal. These were asked to submit all consecutively collected pneumococci causing infections to the central laboratory. Although the laboratories were contacted periodically to submit the isolates to the central laboratory, no audit was performed to ensure compliance, which may be variable in this type of study. The identification of all isolates as *Streptococcus pneumoniae* was confirmed by colony morphology and hemolysis on blood agar plates, optochin susceptibility and bile solubility.

The isolates included in this study were recovered from sputum, bronchial secretions or bronchoalveolar lavage of adult patients (≥18 yrs) with a presumptive diagnosis of pneumonia between 2012 and 2015. Isolates were not included when pneumococci were simultaneously isolated from blood or another usually sterile product, and when other potential bacterial pathogens besides pneumococci were detected in the sample (such as *Haemophilus influenzae*, which was also frequently detected). Only one isolate from each patient in each year was considered.

### Serotyping and antimicrobial susceptibility testing

Serotyping was performed by the standard capsular reaction test using the chessboard system and specific sera (Statens Serum Institut, Copenhagen, Denmark) [23]. Serotypes were classified into vaccine serotypes, i.e., those included in PCV7 (serotypes 4, 6B, 9V, 14, 18C, 19F, 23F), in PCV13 (all PCV7 serotypes and the additional serotypes present only in PCV13, addPCV13: 1, 3, 5, 6A, 7F and 19A), in PPV23 (all PCV13 serotypes, except serotype 6A, and the additional serotypes present only in PPV23, addPPV23: 2, 8, 9N, 10A, 11A, 12F, 15B, 17F, 20, 22F, and 33F) and non-vaccine serotypes (NVT, including all other serotypes). Given the high frequency of spontaneous switching between serotypes 15B and 15C we have opted to group isolates with these serotypes into a single group. Due to difficulties in phenotypically distinguishing isolates of serotype 25A and serogroup 38 and of serogroup 29 and serotype 35B these were also grouped together into the 25A/38 and 29/35B groups. The isolates that were not typable with any of the complete set of sera were considered non-typable (NT).

Minimum inhibitory concentrations (MICs) for penicillin and cefotaxime were determined using Etest strips (Biomérieux, Marcy-L’Etoile, France). Unless otherwise stated, the results were interpreted using the Clinical and Laboratory Standards Institute (CLSI) recommended breakpoints prior to 2008 [24], corresponding to the current breakpoints of oral penicillin V allowing the comparison with previously published data. According to these criteria, intermediate resistance to penicillin is defined as MIC 0.12–1.0 μg/ml and high-level resistance as MIC≥2.0 μg/ml. Isolates that fell into either one of these classes were designated penicillin non-susceptible (PNSP). The interpretation according to the current CLSI guidelines was also performed [25]. According to these criteria, for non-meningitis cases, intermediate resistance to penicillin is defined as MIC between 2–8 μg/ml and high-level resistance as MIC>8 μg/ml. Susceptibility to cefotaxime was defined as MIC≤1.0 μg/ml. The Kirby-Bauer disk diffusion assay was used to determine susceptibility to levofloxacin, erythromycin, clindamycin, chloramphenicol, trimethoprim/sulfamethoxazole, tetracycline, vancomycin and linezolid, according to the CLSI recommendations and interpretative criteria [25]. Macrolide resistance phenotypes were identified using a double disc test with erythromycin and clindamycin, as previously described [14]. The MLS_B_ phenotype (resistance to macrolides, lincosamides and streptogramin B) was defined as the simultaneous resistance to erythromycin and clindamycin, while the M phenotype (resistance to macrolides) was defined as non-susceptibility only to erythromycin.

### Statistical analysis

Sample diversity was measured using Simpson’s index of diversity (SID) and the respective 95% confidence intervals (CI95%) [26]. To compare two sets of partitions the Adjusted Wallace (AW) coefficients were calculated [26] using the online tool available at www.comparingpartitions.info. Differences were evaluated by the Fisher exact test with the false discovery rate (FDR) correction for multiple testing [27] or the Chi-squared test, and the Cochran-Armitage test was used for trends. A p<0.05 was considered significant for all tests.

## Results

### Serotype distribution

A total of 1435 isolates were collected from adults with non-invasive pneumococcal pneumonia: n = 368 in 2012, n = 319 in 2013, n = 311 in 2014 and n = 437 in 2015. Stratifying by age group, 339 isolates (23.6%) were recovered from patients 18–49 years old, 382 (26.6%) from patients 50–64 years old and 714 (49.8%) from patients ≥65 years old. Most of the isolates were recovered from sputum (n = 787, 54.8%), 531 (37.0%) were recovered from bronchial secretions and 117 (8.2%) were recovered from bronchoalveolar lavage fluid. A total of 50 different serotypes were detected. The most frequent serotypes, which accounted for 52% of the isolates, were serotypes 3 (n = 196, 13.7%), 11A (n = 120, 8.4%), 19F (n = 85, 5.9%), 23A (n = 67, 4.7%), 6C (n = 64, 4.5%), 19A (n = 58, 4.0%), 23B (n = 56, 3.9%), 9N (n = 52, 3.6%) and NT isolates (n = 50, 3.5%).

The S1 Fig represent the number of isolates expressing serotypes included in PCVs, the addPPV23, and the number of isolates expressing NVTs, respectively, stratified by age group. Serotype diversity was high—SID = 0.952, CI95%: 0.948–0.956 –with no difference between SIDs of different years. No individual serotype (n>15 isolates) showed differences in age distribution, statistically supported after FDR correction.

**Fig 1:**
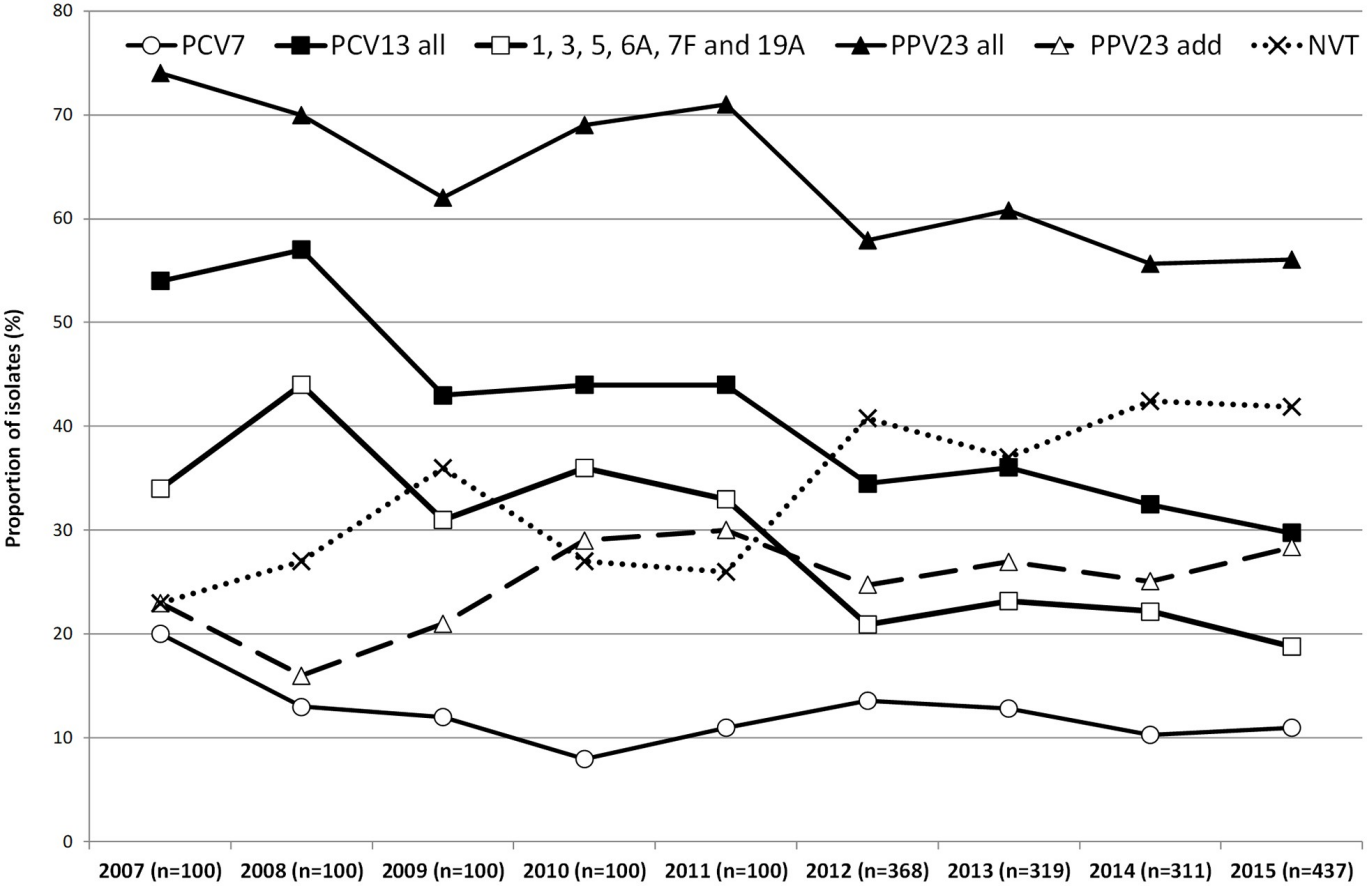
Proportion of isolates expressing serotypes included in each of the pneumococcal vaccines causing non-invasive pneumococcal pneumonia in adult patients (≥18 years) in Portugal, 2007–2015. The data up to 2011 were presented previously [14].

Fig 1 shows the proportion of potentially vaccine preventable NIPP during the study period and, for comparison purposes, also the previously published data from 2007–2011, since these years represent the late post-PCV7 period (2007–2009) and the first two years of PCV13 use in children (2010–2011) [14]. Considering the evolution during the current study period only (2012–2015), there was a decline in the proportion of NIPP caused by PCV13 serotypes, from 34.5% in 2012 to 29.7% in 2015, but this was not statistically supported (p = 0.090). This decline was associated with slight and non-significant decreases in both the proportion of NIPP caused by PCV7 serotypes (from 13.6% to 11.0%, p = 0.177) and addPCV13 (from 20.9% to 18.8%, p = 0.377). In contrast, there was a non-significant increase in the proportion of NIPP caused by addPPV23 (from 24.7% in 2012 to 28.4% in 2015, p = 0.325), while the proportion of NIPP caused by NVTs remained relatively stable from 2012 to 2015 (40.8% vs 41.9%, respectively, p = 0.460).

We then evaluated possible serotype trends since 2010 when PCV13 started being used in children through the private market. The overall proportion of PCV13 serotypes declined from 44.0% in 2010 to 29.7% in 2015 (p<0.001), while that of addPCV13 decreased from 36.0% in 2010 to 18.8% in 2015 (p<0.001). This was accompanied by an increase of NVTs from 27.0% in 2010 to 41.9% in 2015 (p = 0.002). The PCV7 and addPPV23 serotypes remained relatively stable.

Table 1 shows the evolution of the individual serotypes causing NIPP in adults during the current study period (2012–2015). Only serotype 25A/38 significantly changed its proportion after FDR correction (from 1.9% in 2012 to 0.0% in 2015, p = 0.001). When considering the evolution of individual serotypes since 2007 (Table 1 and S1 Table), only four serotypes significantly changed their proportion after FDR correction (S2 Fig), which were serotypes 3 (declined from 22.0% in 2007 to 12.1% in 2015, p<0.001), 19A (declined from 6.0% in 2007 to 3.2% in 2015, p = 0.003), 7F (declined from 3.0% in 2007 to 1.1% in 2015, p = 0.004) and 35F (increased from 0% in 2007 to 3.2% in 2015, p = 0.003). The declines in proportion of serotypes 3 and 19A showed important yearly fluctuations and these were also found for several other serotypes (Table 1 and S2 Table).

**Table 1:** Serotypes of the isolates responsible for non-invasive pneumococcal pneumonia in adult patients (≥18 years), 2012–2015.

Serotype	No. of isolates (%)				CA^a^
	2012	2013	2014	2015	2012–2015
PCV13					
1	2 (0.5)	0 (0)	0 (0)	1 (0.2)	0.399
3	48 (13.0)	54 (16.9)	41 (13.2)	53 (12.1)	0.408
4	1 (0.3)	1 (0.3)	2 (0.6)	3 (0.7)	0.329
5	0 (0)	0 (0)	0 (0)	0 (0)	-
6A	5 (1.4)	7 (2.2)	6 (1.9)	9 (2.1)	0.547
6B	7 (1.9)	4 (1.3)	4 (1.3)	6 (1.4)	0.578
7F	5 (1.4)	4 (1.3)	4 (1.3)	5 (1.1)	0.800
9V	0 (0)	5 (1.6)	0 (0)	0 (0)	0.275
14	12 (3.3)	6 (1.9)	6 (1.9)	10 (2.3)	0.426
18C	4 (1.1)	1 (0.3)	1 (0.3)	1 (0.2)	0.106
19A	17 (4.6)	9 (2.8)	18 (5.8)	14 (3.2)	0.645
19F	22 (6.0)	22 (6.9)	15 (4.8)	26 (5.9)	0.746
23F	4 (1.1)	2 (0.6)	4 (1.3)	2 (0.5)	0.483
PPV23 only					
8	7 (1.9)	10 (3.1)	6 (1.9)	16 (3.7)	0.222
9N	11 (3.0)	13 (4.1)	16 (5.1)	12 (2.7)	0.942
10A	11 (3.0)	6 (1.9)	5 (1.6)	10 (2.3)	0.519
11A	29 (7.9)	29 (9.1)	22 (7.1)	40 (9.2)	0.703
12F	0 (0)	0 (0)	0 (0)	0 (0)	-
15B/C	6 (1.6)	11 (3.4)	7 (2.3)	10 (2.3)	0.807
17F	8 (2.2)	4 (1.3)	8 (2.6)	4 (0.9)	0.319
20	5 (1.4)	5 (1.6)	6 (1.9)	8 (1.8)	0.557
22F	14 (3.8)	8 (2.5)	6 (1.9)	21 (4.8)	0.448
33F	0 (0)	0 (0)	2 (0.6)	3 (0.7)	0.048
NVT^b^					
6C	19 (5.2)	16 (5.0)	7 (2.3)	22 (5.0)	0.627
23A	24 (6.5)	12 (3.8)	14 (4.5)	17 (3.9)	0.130
23B	15 (4.1)	8 (2.5)	15 (4.8)	18 (4.1)	0.631
NT	9 (2.4)	8 (2.5)	16 (5.1)	17 (3.9)	0.123
15A	10 (2.7)	9 (2.8)	8 (2.6)	16 (3.7)	0.465
31	15 (4.1)	7 (2.2)	14 (4.5)	12 (2.7)	0.587
16F	7 (1.9)	10 (3.1)	3 (1.0)	20 (4.6)	0.070
29/35B	12 (3.3)	6 (1.9)	6 (1.9)	10 (2.3)	0.426
35F	5 (1.4)	3 (0.9)	6 (1.9)	14 (3.2)	0.033
34	3 (0.8)	8 (2.5)	5 (1.6)	8 (1.8)	0.445
21	3 (0.8)	6 (1.9)	7 (2.3)	8 (1.8)	0.265
24F	4 (1.1)	2 (0.6)	6 (1.9)	10 (2.3)	0.082
33A	6 (1.6)	1 (0.3)	5 (1.6)	0 (0)	0.052
25A/38	7 (1.9)	2 (0.6)	0 (0)	0 (0)	**0.001**
35A	2 (0.5)	3 (0.9)	4 (1.3)	2 (0.5)	0.946
7C	2 (0.5)	2 (0.6)	6 (1.9)	1 (0.2)	0.946
13	2 (0.5)	3 (0.9)	1 (0.3)	1 (0.2)	0.333
37	1 (0.3)	4 (1.3)	1 (0.3)	3 (0.7)	0.802
Others^c^	4 (1.1)	8 (2.5)	8 (2.6)	4 (0.9)	-
Total	368	319	311	437	-

^a^CA, Cochran Armitage test of trend. In bold is the only serotype with significant p-value (p < 0.05) after FDR correction.

^b^NVT, non-vaccine serotypes, i.e., serotypes not included in any of the currently available pneumococcal vaccines.

^c^Only serotypes detected in ≥3 isolates in at least one year are shown; the remaining are grouped together under “Others.”

When analyzing the evolution of individual vaccine serotypes and of vaccine serotype groups in 2012–2015 stratified by age group (Table 2), there were no significant changes after FDR correction. When considering data since 2007, only for serotype 3 and for adults aged ≥65 years old did the change remain statistically supported after FDR correction (serotype 3 declined from 27.5% in 2007 to 11.1% in 2015, p<0.001).

**Table 2:** Number of isolates responsible for non-invasive pneumococcal pneumonia in adult patients (≥18 years), according to vaccine serotype groups and age groups, 2012–2015.

	Serotype Groups^b^	No. isolates (%)			2015	CA^a^
		2012	2013	2014		
18–49 years	PCV7	12 (12.9)	11 (16.4)	8 (10.3)	16 (15.8)	0.782
	1, 5 and 7F	1 (1.1)	1 (1.5)	2 (2.6)	1 (1.0)	0.926
	3, 6A and 19A	19 (20.4)	11 (16.4)	10 (12.8)	20 (19.8)	0.800
	PCV13	32 (34.4)	23 (34.3)	20 (25.6)	37 (36.6)	0.983
	addPPV23	25 (26.9)	15 (22.4)	28 (35.9)	30 (29.7)	0.417
	NVTs	36 (38.7)	29 (43.3)	30 (38.5)	34 (33.7)	0.385
50–64 years	PCV7	12 (14.1)	7 (8.4)	7 (7.4)	8 (6.7)	0.328
	1, 5 and 7F	3 (3.5)	0 (0)	1 (1.1)	1 (0.8)	0.656
	3, 6A and 19A	15 (17.6)	23 (27.7)	23 (24.2)	19 (16.0)	0.095
	PCV13	30 (35.3)	30 (36.1)	31 (32.6)	28 (23.5)	0.026
	PPV23 add	45 (18.8)	50 (27.7)	53 (24.2)	65 (32.8)	0.082
	NVTs	39 (45.9)	30 (36.1)	41 (24.2)	52 (32.8)	0.531
≥65 years	PCV7	26 (13.7)	23 (13.6)	17 (12.3)	24 (1.1)	0.381
	1, 5 and 7F	3 (1.6)	3 (1.8)	1 (0.7)	4 (1.8)	0.976
	3, 6A and 19A	36 (18.9)	36 (21.3)	32 (23.2)	37 (17.1)	0.665
	PCV13	65 (34.2)	62 (36.7)	50 (36.2)	65 (30.0)	0.332
	PPV23 add	50 (26.3)	48 (28.4)	27 (19.6)	55 (25.3)	0.078
	NVTs	75 (39.5)	59 (34.9)	61 (44.2)	97 (44.7)	0.125

^a^ CA, Cochran Armitage test of trend.

^b^ PCV7, serotypes included in the 7-valent pneumococcal conjugate vaccine. PCV13, serotypes included in the 13-valent pneumococcal conjugate vaccine. addPPV23, the additional 11 serotypes present in the 23-valent pneumococcal polysaccharide vaccine but absent from PCV13. NVTs, serotypes not included in any of the currently available pneumococcal vaccines.

### Antimicrobial susceptibility

Susceptibility to the tested antimicrobials between 2012 and 2015 stratified by the age groups considered is summarized in Table 3. When considering all isolates, a total of n = 258 isolates (18.0%) were classified as PNSP of which n = 229 (88.8%) expressed low-level resistance and n = 29 (11.2%), high-level resistance. According to the current CLSI guidelines for parental penicillin in non-meningitis cases [25], only n = 15 isolates (1.0%) would have been considered PNSP, with only 2 of these expressing high-level resistance. A total of n = 311 isolates (21.7%) were classified as erythromycin resistant pneumococci (ERP). Of these, n = 246 isolates (79.1%) expressed the MLS_B_ phenotype, while the remaining (n = 65, 20.9%) presented the M phenotype. A total of 12.3% (n = 176) of the isolates were simultaneously non-susceptible to penicillin and resistant to erythromycin (EPNSP).

**Table 3:** Antimicrobial resistance of the isolates responsible for non-invasive pneumococcal pneumonia in adult patients (≥18 years) in Portugal, 2012–2015.

	No. resistant isolates (%)^a^		
	18–49 years (n = 339)	50–64 years (n = 382)	≥65 years (n = 714)
PEN^b^	57 (16.8)	70 (18.3)	131 (18.3)
MIC90	0.19	0.19	0.38
MIC50	0.012	0.012	0.012
CTX	7 (2.1)	7 (1.8)	4 (0.6)
MIC90	0.25	0.25	0.38
MIC50	0.015	0.016	0.016
LEV	2 (0.6)	3 (0.8)	16 (2.2)
ERY	73 (21.5)	70 (18.3)	168 (23.5)
CLI	56 (16.5)	58 (15.2)	136 (19.0)
CHL	7 (2.1)	7 (1.8)	4 (0.6)
SXT	57 (16.8)	66 (17.3)	104 (14.6)
TET	59 (17.4)	55 (14.4)	115 (16.1)

^a^PEN, penicillin; CTX, cefotaxime; LEV, levofloxacin; ERY, erythromycin; CLI, clindamycin; CHL, chloramphenicol; SXT, trimethoprim/sulfamethoxazole; TET, tetracycline. All isolates were susceptible to vancomycin and linezolid.

^b^Non-susceptibitily to penicillin was determined using the CLSI breakpoints prior to 2008 [24].

There were no significant variations in antimicrobial resistance during the current study period (2012–2015), nor were there significant changes in antimicrobial resistance when considering NIPP from 2007 [14]. Although with moderate overall AW values [the AW for serotype to PNSP was 0.441 (CI95%: 0.386–0.496) and the AW for serotype to ERP was 0.443 (CI95%: 0362–0.524)], there was an association between certain serotypes and antimicrobial resistance (S1 Fig). The serotypes that were positively associated with PNSP after FDR correction were serotypes 6C, 14, 15A, 19F, 19A and 23F. Among these, serotypes 19F (15.5%), 14 (12.4%), 6C (12.0%) and 19A (11.6%) accounted for half of all PNSP. The serotypes which were positively associated with ERP after FDR correction were serotypes 6B, 6C, 14, 15A, 19F, 19A, 33A and 35A, of which serotypes 19F (20.3%), 19A (10.6%), 6C (9.6%) and 15A (8.0%) accounted for half of all ERP. The PCV7, PCV13 and PPV23 serotypes accounted for 33.7%, 47.3% and 53.5% of PNSP, respectively, and 33.4%, 49.5% and 54.3% of ERP, respectively.

### Discussion

The present study documented a decline of PCV13 serotypes in adult NIPP in the post-PCV13 period. This occurred mostly in 2011–2012 but continued, albeit more moderately, in recent years, from 44.0% in 2010 to 29.7% in 2015. It was also noted that during 2007–2015 there were several important yearly fluctuations in the proportion of individual serotypes, both among PCV13 and non-PCV13 serotypes (Table 1 and S1 Table). This suggests that variations of the PCV13 serotypes in the post-PCV13 period in adult NIPP could be the result of, not only the herd protection conferred by childhood vaccination with PCV13, but also of temporal trends, which had been documented in adult NIPP in Portugal previously [14].

The evolution of PCV13 serotypes in adult NIPP from 2010 onwards (when PCV13 was being used for children vaccination in the private market) was different from the one previously found for adult IPD in Portugal in a similar period [6]. While in NIPP the sharpest decrease in addPCV13 serotypes in the post-PCV13 period occurred from 2011 to 2012, in IPD this occurred only from 2012 to 2013. Although the decrease of addPCV13 serotypes in adult NIPP may have been also influenced by temporal trends, the sustained lower values found from 2013 onwards suggest an important contribution of herd protection resulting from PCV13 childhood vaccination.

Serotypes 3 and 19A had a major influence in the decrease of PCV13 serotypes in adult NIPP in the post-PCV13 period (2010–2015), although these changes were also most significant in the first years (Table 1 and S1 Table). Similarly, in adult IPD two serotypes accounted for most of the decline in the prevalence of PCV13 serotypes in the post-PCV13 period, but in this case these were serotypes 7F and 19A [6]. In contrast with the declines of serotypes 3 and 19A in adult NIPP, the decreases of serotypes 7F and 19A in adult IPD were more pronounced and sustained.

Serotype 3 has been the dominant serotype in adult NIPP and IPD in Portugal, both before and after the introduction of PCV13 for children [6,14]. The decline in serotype 3 in NIPP is surprising because this serotype did not show major changes in adult IPD in the post-PCV13 period in Portugal [6] nor in other countries [7,8,28–30]. However, reductions in incidence of serotype 3 NIPP was reported in other studies, including a study from England [10]. The reduced efficacy of PCV13 in preventing pediatric complicated pneumonias caused by serotype 3 [17] and the use of PCV13 outside of the national immunization program with somewhat modest uptake, raise the possibility of continued circulation of this serotype in carriage, potentially explaining its persistence in disease. Since serotype 3 is heterogeneous in its invasive disease potential, meaning that there are different clones expressing this serotype that differ in their capacity to cause invasive disease [31], it is possible that more invasive clones of serotype 3 have increased post-PCV use for reasons that remain unknown. In adult IPD, there was an expansion of the multilocus sequence type clonal complex CC180 among isolates expressing serotype 3 [32], prior to the use of PCV13 in children, but no information is available in the post-vaccine period.

Serotype 19A emerged in Portugal in the late post-PCV7 period, to become one of the most important serotypes in both adult NIPP [14] and IPD [5,6,33]. A decrease of serotype 19A in adult IPD and in NIPP in the post-PCV13 period was documented not only for Portugal but for other countries [7,8,10,11,30]. Given the compelling evidence of herd protection in adult IPD resulting from PCV13 use in children in serotype 19A, the lack of a more significant reduction of serotype 19A in adult NIPP in the post-PCV13 period could be due to a particular propensity of this serotype to cause NIPP. A clearer picture of the impact of PCV13 use in children in reducing the importance of serotypes 3 and 19A in adult NIPP may only be provided by further studies following the epidemiology of adult NIPP after the inclusion of PCV13 in the national immunization plan.

A decrease in serotype 7F was also detected but its contribution to the reduction of PCV13 serotypes in NIPP was minor since this serotype was an uncommon cause of NIPP in the pre-PCV13 period.

Contrasting with the declining trend of PCV13 serotypes, no significant trend was seen for PCV7 serotypes in adult NIPP and this was mostly due to the persistence of serotype 19F, which occurred in 49% of the isolates expressing a PCV7 serotype in 2012–2015. Despite being targeted by all PCVs available to date, serotype 19F remained common in nasopharyngeal carriage of children in Portugal in the late post-PCV7 period [34] and in the post-PCV13 period [35], including among vaccinated children. The inability of the PCVs to eliminate this serotype from carriage in children, at least in a non-universal coverage scenario, together with its likely intrinsic propensity to cause NIPP rather than IPD [14] as was also shown here, may have contributed to why this serotype remained the third most frequent cause of adult NIPP in the post-PCV13 period in Portugal.

The decrease of PCV13 serotypes in the post-PCV13 period was accompanied by an increase in the proportion of NVTs, while the addPPV23 serotypes remained relatively stable. However, among the NVTs, only one serotype was clearly emerging (serotype 35F) and only in the last year of the study period. Most of the remaining increase in NVTs was based in increases in the proportion of serotypes 16F, 24F and NTs (Table 1 and S1 Table), which were not significant if considered independently. This contrasts with results from adult IPD, in which there were several non-PCV13 emerging serotypes (serotypes 8, 22F, 20 and 15A), most of them included in PPV23 [6]. These differences are not surprising, since isolates responsible for adult NIPP and adult IPD are known to have different serotype distributions [14,15].

When comparing the serotype distribution of isolates causing adult NIPP in 2012–2014 with the serotype distribution of isolates causing adult IPD in the same period (S2 Table), serotypes 11A, 19F, 23A, 23B, 31, NT, 17F, 6A, 21 and 37 (ranked by their frequency in NIPP) were significantly associated with NIPP, while serotypes 8, 19A, 22F, 14, 7F, 20, 1, 4 and 12B (ranked by their frequency in IPD) were significantly associated with IPD. Most of these associations had been already recognized in the pre-PCV13 period [14], while the new associations in adult IPD reflect mainly the emerging serotypes in the post-PCV13 period.

While antimicrobial resistance declined in adult IPD in the post-PCV13 period, no decline was found for adult NIPP in this study. In NIPP, the small decrease in proportion of the mostly antimicrobial resistant serotype 19A isolates, was balanced by an increase of NT isolates, which were also associated with antimicrobial resistance. NTs were found to be frequent colonizers of the nasopharynx of children in the post-PCV13 period [34] and were more frequently found in NIPP than in IPD (S2 Table). The stability of PCV7 serotypes in the post-PCV13 period also helped maintaining antimicrobial resistance rates in adult NIPP [14].

The study presented has the limitations discussed previously [14]. These include the possibility that some of the isolates we identified as being responsible for NIPP were in fact causing bacteremic pneumonia or reflected colonization and not disease. Despite the general recommendation that both blood and respiratory tract samples should be collected for the etiologic diagnosis of pneumonia, we cannot guarantee that this was done in all cases. However, we consider these to account, at most, for a small fraction of the isolates and therefore not to introduce a significant bias. Moreover, the distinct serotype distribution found in this study for IPD and NIPP, strongly argues against this possibility. Since our study is laboratory-based, it was not designed to collect information important to assess the severity of the infections caused by the different serotypes (e.g. hospitalization, ICU admission, 30-day mortality). However, this does not compromise our approach of comparing the serotype distribution of IPD and NIPP cases. Our temporal analyses were based on previously published data reporting the characteristics of a random sample of 100 isolates per year [14]. Since not all available isolates before 2012 were characterized, it is possible that some of the changes in the serotype distribution occurring from 2011 to 2012 are due to this sampling process.

In this study it was found that the overall proportion of PCV13 serotypes decreased only moderately in adult NIPP in the post-PCV13 period. In 2015, 30% of NIPP was due to PCV13 serotypes and 28% was due to the addPPV23 serotypes, highlighting the potential role of vaccination in disease prevention. However, the inclusion of PCV13 in the national immunization program for children in 2015 and the anticipated declines in at least some of the PCV13 serotypes due to herd effect, raise important issues regarding the cost-effectiveness of a universal adult vaccination program. However, because the magnitude and timeframe of this herd effect remains poorly defined, particularly in NIPP, further surveillance is essential to document future trends in pneumococcal serotype prevalence in adult NIPP, as these seem to differ from adult IPD.

## Supporting information

S1 Fig Number of isolates expressing each serotype causing non-invasive pneumococcal pneumonia in adult patients (≥18 yrs), Portugal, 2012–2015. The number of isolates expressing each serotype in each of the age groups considered is indicated. Isolates recovered from patients 18–49 years are indicated by black triangles. Isolates recovered from patients 50–64 years are indicated by open squares. Isolates recovered from patients ≥ 65years are indicated by open circles. Isolates presenting both erythromycin resistance and penicillin non-susceptibility (EPNSP) are represented by closed black bars. Penicillin non-susceptible isolates (PNSP) are indicated by dark hatched bars. Erythromycin resistant pneumococci (ERP) are indicated by light hatched bars. Isolates susceptible to both penicillin and erythromycin are represented by white open bars. **Panel A—Serotypes included in conjugate vaccines**. The serotypes included in the seven-valent conjugate vaccine (PCV7) and in the 13-valent conjugate vaccine (PCV13) are indicated by the arrows. NVT, non-vaccine serotypes; addPPV23, the additional serotypes included in the 23-valent polysaccharide vaccine but not included in PCV13. **Panel B—Additional serotypes included in the 23-valent polysaccharide vaccine but not included in the 13-valent conjugate vaccine**. Out of the 11 addPPV23 serotypes only serotype 2 was not found in our collection. **Panel C—Serotypes not included in any pneumococcal vaccine** NT, non-typable. Isolates expressing serotypes 25A and 38 and serotypes 29 and 35B could not be distinguished phenotypically and are represented together. Only serotypes including n>3 isolates are discriminated, all remaining serotypes are grouped together under the “Others” category grouping isolates of serotypes: 10B, 12B, 17A, 18A (n = 3 each); 10F, 11F, 11B and 47F (n = 2 each) and 28A, 35C, 36 and 42 (n = 1 each).
(PDF)

S2 Fig Isolates expressing serotypes that changed in proportion after FDR correction causing non-invasive pneumococcal pneumonia in adult patients (≥18 years) in Portugal, 2007–2015. The data up to 2011 were presented previously [14].
(PDF)

S1 Table Serotypes of the isolates responsible for non-invasive pneumococcal pneumonia in adult patients (≥18 years), 2007–2011. These data were presented previously [14].
(PDF)

S2 Table Serotype distribution of the isolates causing non-invasive pneumococcal pneumonia and invasive pneumococcal disease in adults in Portugal (2012–2014). (PDF)
