# Supplementary material for: Conjugate vaccine serotypes persist as major causes of non-invasive pneumococcal pneumonia in Portugal despite declines in serotypes 3 and 19A (2012-2015)
Source: PLoS One. 2018 Nov 2;13(11):e0206912. doi: 10.1371/journal.pone.0206912 (PMC6214563; doi:10.1371/journal.pone.0206912)
Supplement: S1 Fig — The number of isolates expressing each serotype in each of the age groups considered is indicated. Isolates recovered from patients 18–49 years are indicated by black triangles. Isolates recovered from patients 50–64 years are indicated by open squares. Isolates recovered from patients ≥ 65years are indicated by open circles. Isolates presenting both erythromycin resistance and penicillin non-susceptibility (EPNSP) are represented by closed black bars. Penicillin non-susceptible isolates (PNSP) are indicated by dark hatched bars. Erythromycin resistant pneumococci (ERP) are indicated by light hatched bars. Isolates susceptible to both penicillin and erythromycin are represented by white open bars. Panel A—Serotypes included in conjugate vaccines. The serotypes included in the seven-valent conjugate vaccine (PCV7) and in the 13-valent conjugate vaccine (PCV13) are indicated by the arrows. NVT, non-vaccine serotypes; addPPV23, the additional serotypes included in the 23-valent polysaccharide vaccine but not included in PCV13. Panel B—Additional serotypes included in the 23-valent polysaccharide vaccine but not included in the 13-valent conjugate vaccine. Out of the 11 addPPV23 serotypes only serotype 2 was not found in our collection. Panel C—Serotypes not included in any pneumococcal vaccine NT, non-typable. Isolates expressing serotypes 25A and 38 and serotypes 29 and 35B could not be distinguished phenotypically and are represented together. Only serotypes including n>3 isolates are discriminated, all remaining serotypes are grouped together under the “Others” category grouping isolates of serotypes: 10B, 12B, 17A, 18A (n = 3 each); 10F, 11F, 11B and 47F (n = 2 each) and 28A, 35C, 36 and 42 (n = 1 each). (PDF) [file pone.0206912.s001.pdf]

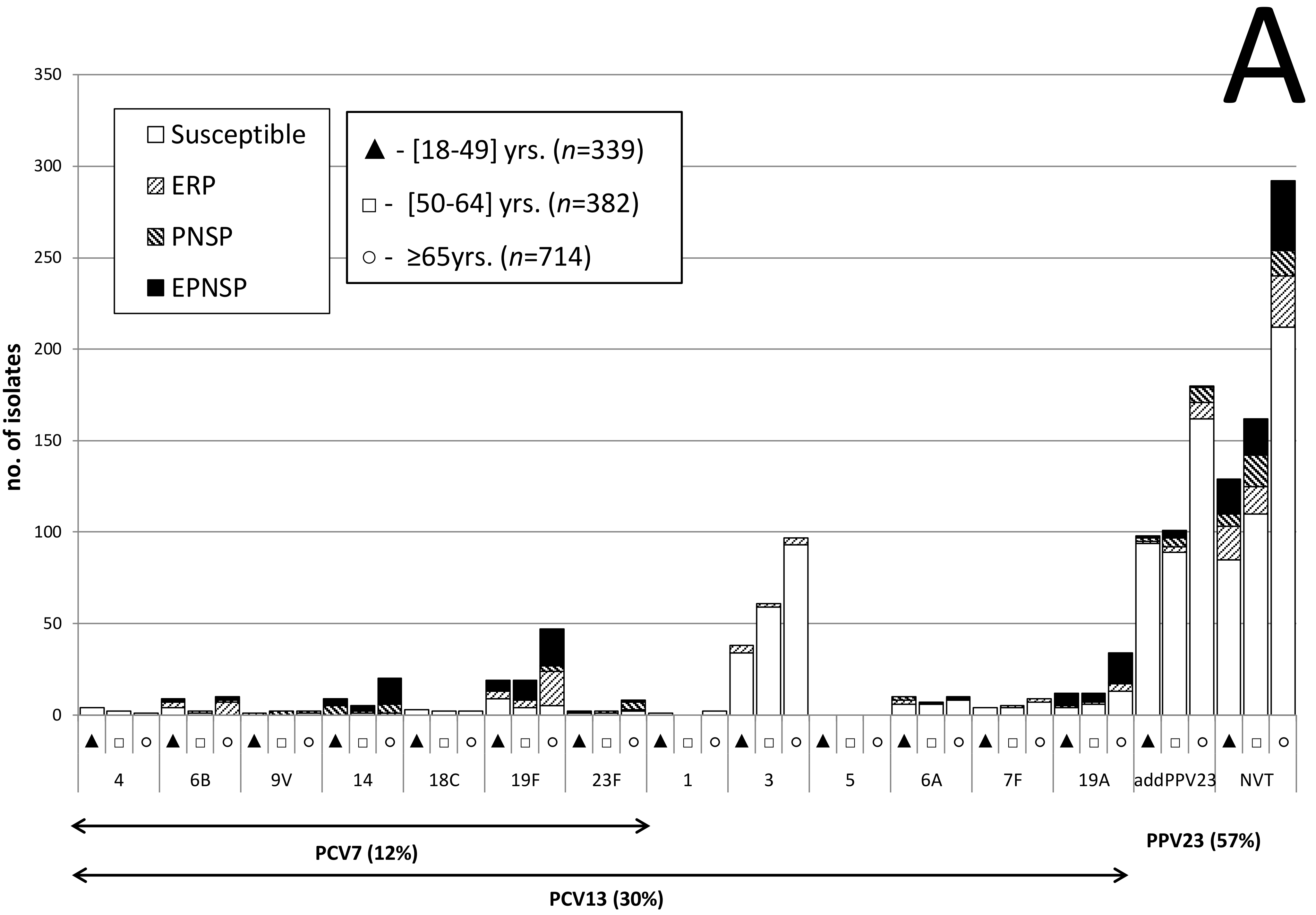

A
350
300
250
200
150
100
50
0
no. of isolates
Susceptible
ERP
PNSP
EPNSP
▲ - [18-49] yrs. (n=339)
□ - [50-64] yrs. (n=382)
○ - ≥65yrs. (n=714)
4
6B
9V
14
18C
19F
23F
1
3
5
6A
7F
19A
addPPV23
NVT
PCV7 (12%)
PPV23 (57%)
PCV13 (30%)

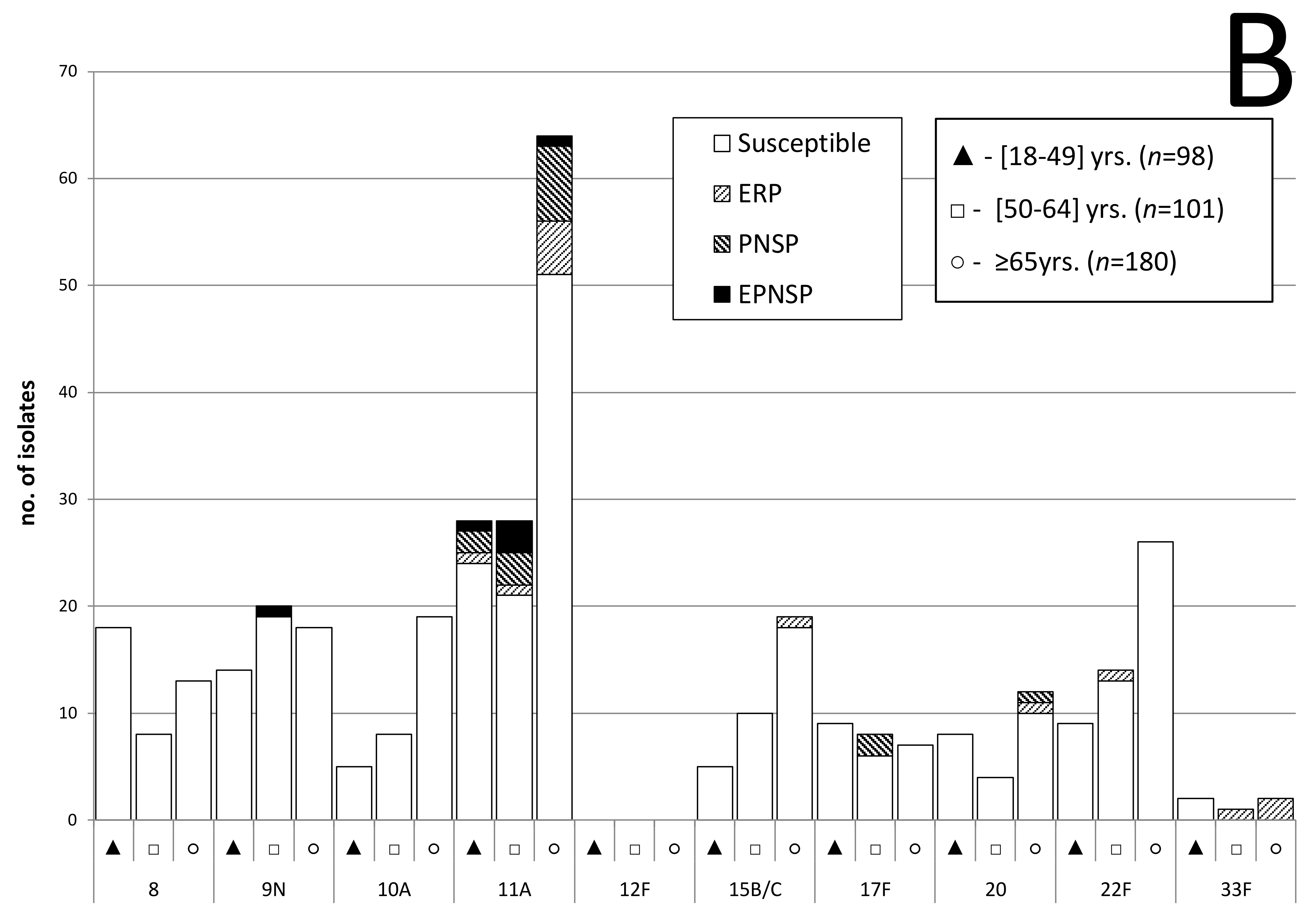
B
Susceptible
ERP
PNSP
EPNSP
▲ - [18-49] yrs. (n=98)
□ - [50-64] yrs. (n=101)
○ - ≥65yrs. (n=180)
no. of isolates
0
10
20
30
40
50
60
70
8
9N
10A
11A
12F
15B/C
17F
20
22F
33F

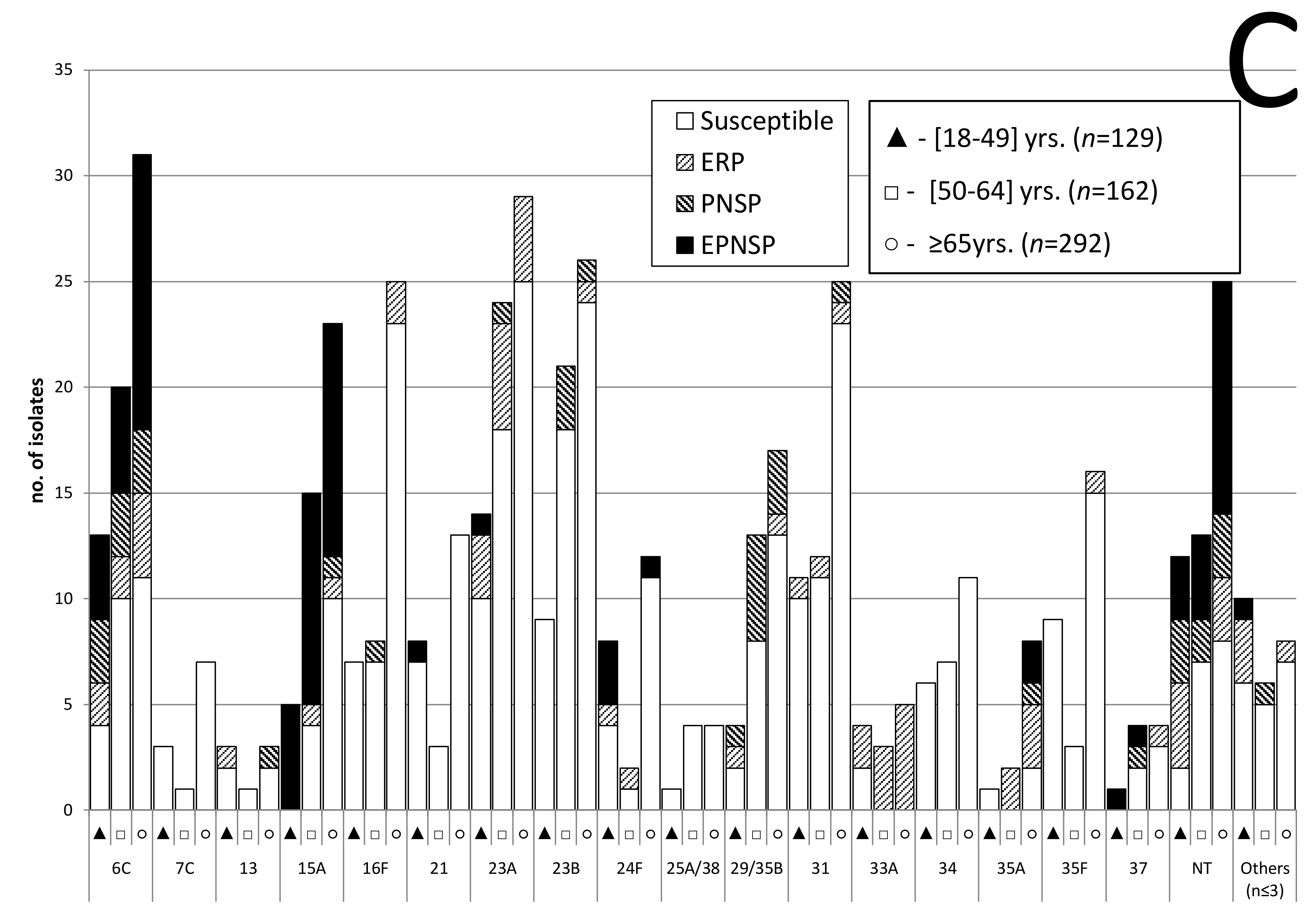
C
Susceptible
ERP
PNSP
EPNSP
▲ - [18-49] yrs. (n=129)
□ - [50-64] yrs. (n=162)
○ - ≥65yrs. (n=292)
no. of isolates
0
5
10
15
20
25
30
35
6C
7C
13
15A
16F
21
23A
23B
24F
25A/38
29/35B
31
33A
34
35A
35F
37
NT
Others (n≤3)

S1 Fig. Number of isolates expressing each serotype causing non-invasive pneumococcal pneumonia in adult patients (≥18 yrs), Portugal, 2012-2015. The number of isolates expressing each serotype in each of the age groups considered is indicated. Isolates recovered from patients 18-49 years are indicated by black triangles. Isolates recovered from patients 50-64 years are indicated by open squares. Isolates recovered from patients ≥ 65years are indicated by open circles. Isolates presenting both erythromycin resistance and penicillin non-susceptibility (EPNSP) are represented by closed black bars. Penicillin non-susceptible isolates (PNSP) are indicated by dark hatched bars. Erythromycin resistant pneumococci (ERP) are indicated by light hatched bars. Isolates susceptible to both penicillin and erythromycin are represented by white open bars. **Panel A - Serotypes included in conjugate vaccines.** The serotypes included in the seven-valent conjugate vaccine (PCV7) and in the 13-valent conjugate vaccine (PCV13) are indicated by the arrows. NVT, non-vaccine serotypes; addPPV23, the additional serotypes included in the 23-valent polysaccharide vaccine but not included in PCV13. **Panel B - Additional serotypes included in the 23-valent polysaccharide vaccine but not included in the 13-valent conjugate vaccine.** Out of the 11 addPPV23 serotypes only serotype 2 was not found in our collection. **Panel C - Serotypes not included in any pneumococcal vaccine.** NT, non-typable. Isolates expressing serotypes 25A and 38 and serotypes 29 and 35B could not be distinguished phenotypically and are represented together. Only serotypes including n>3 isolates are discriminated, all remaining serotypes are grouped together under the "Others" category grouping isolates of serotypes: 10B, 12B, 17A, 18A (n=3 each); 10F, 11F, 11B and 47F (n=2 each) and 28A, 35C, 36 and 42 (n=1 each)
