# Supplementary material for: Conjugate vaccine serotypes persist as major causes of non-invasive pneumococcal pneumonia in Portugal despite declines in serotypes 3 and 19A (2012-2015)
Source: PLoS One. 2018 Nov 2;13(11):e0206912. doi: 10.1371/journal.pone.0206912 (PMC6214563; doi:10.1371/journal.pone.0206912)
Supplement: S2 Fig — The data up to 2011 were presented previously [14]. (PDF) [file pone.0206912.s002.pdf]

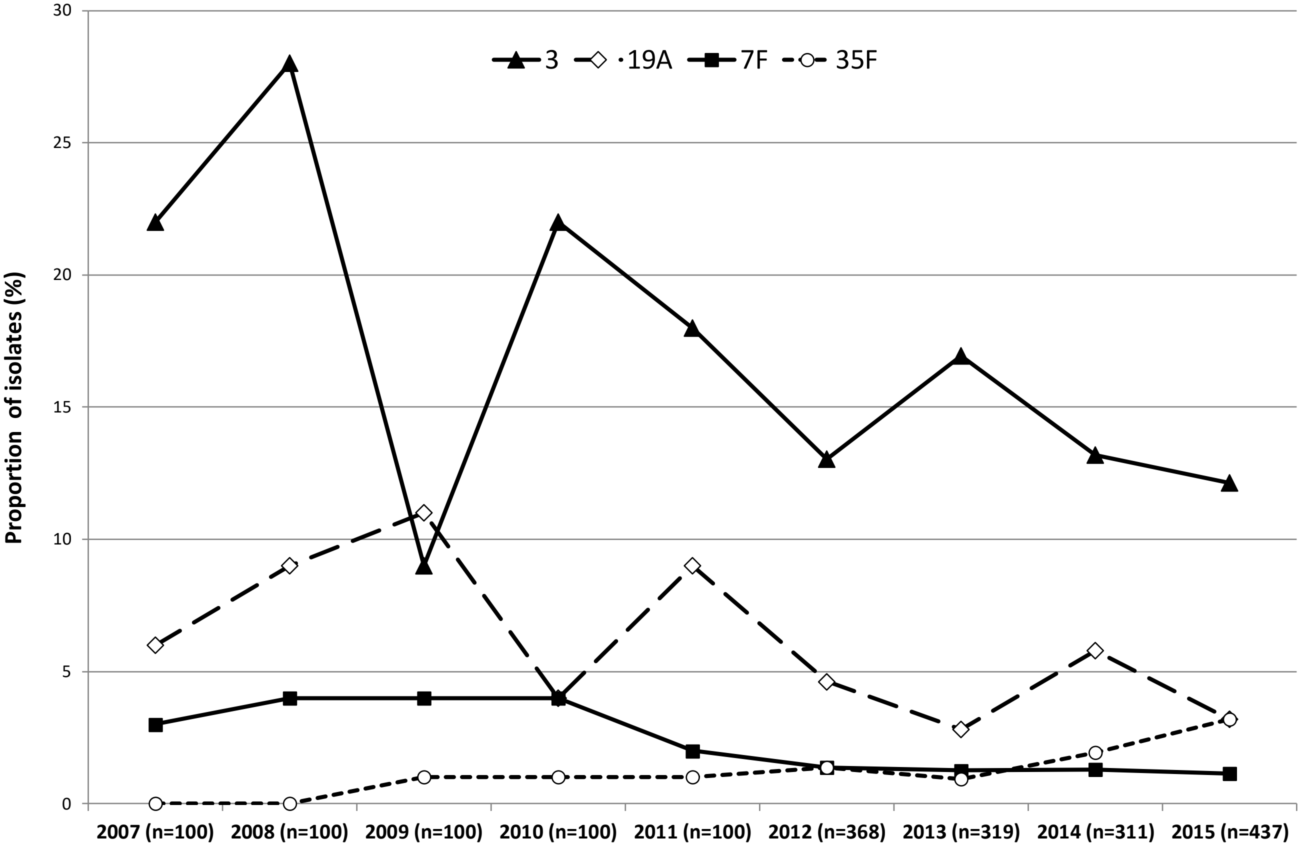

S2 Fig. Isolates expressing serotypes that changed in proportion after FDR correction causing non-invasive pneumococcal pneumonia in adult patients (≥18 years) in Portugal, 2007–2015. The data up to 2011 were presented previously [14].
