# Supplementary material for: Conjugate vaccine serotypes persist as major causes of non-invasive pneumococcal pneumonia in Portugal despite declines in serotypes 3 and 19A (2012-2015)
Source: PLoS One. 2018 Nov 2;13(11):e0206912. doi: 10.1371/journal.pone.0206912 (PMC6214563; doi:10.1371/journal.pone.0206912)
Supplement: S1 Table — These data were presented previously [14]. (PDF) [file pone.0206912.s003.pdf]

S1 Table. Serotypes of the isolates responsible for non-invasive pneumococcal pneumonia in adult patients (≥18 years), 2007–2011. These data were presented previously [14].

Serotype[a]	No. of isolates					CA[b]
	2007	2008	2009	2010	2011	2007-2015
PCV13						
1	0	0	3	2	1	0.039
3	22	28	9	22	18	**<0.001**
4	1	0	0	1	1	0.776
5	0	0	2	0	0	0.041
6A	3	3	2	4	3	0.342
6B	3	1	0	1	0	0.926
7F	3	4	4	4	2	**0.004**
9V	3	0	0	0	0	0.048
14	4	3	4	2	3	0.172
18C	0	2	0	0	0	0.435
19A	6	9	11	4	9	**0.003**
19F	6	3	7	4	6	0.682
23F	3	4	1	0	1	0.011
addPPV23						
8	3	0	3	2	5	0.316
9N	1	0	4	6	2	0.184
10A	2	0	2	1	1	0.394
11A	9	8	4	10	7	0.582
12F	0	0	0	0	1	0.555
15B/C	1	2	4	3	3	0.864
17F	1	2	0	0	3	0.734
20	0	1	1	0	2	0.087
22F	6	3	3	7	6	0.398
33F	0	0	0	0	0	0.035
NVT						
6C	2	4	10	5	2	0.786
23A	3	4	3	1	2	0.440
23B	2	2	4	3	7	0.308
NT	1	3	2	1	2	0.021
15A	2	3	4	2	4	0.632
31	2	1	1	1	0	0.063
16F	2	2	2	3	0	0.097
29/35B	2	1	2	4	2	0.974
35F	0	0	1	1	1	**0.003**
34	0	0	0	1	2	0.023
21	0	0	1	1	0	0.015
24F	2	0	2	0	0	0.133
33A	0	1	2	1	1	0.373
25A/38	1	2	1	0	0	0.024

13	1	0	0	1	1	0.600
Others[c]	3	4	1	2	2	-
Total	100	100	100	100	100	-

[a]NVT, non-vaccine serotypes, i.e., serotypes not included in any of the currently available pneumococcal vaccines.

[b]CA, Cochran Armitage test of trend. In bold are the serotypes with significant p-value ($p<0.05$) after FDR correction.

[c]Only serotypes detected in ≥3 isolates or in at least 2 years are shown; the remaining are grouped together under “Others.”
