# Supplementary material for: Conjugate vaccine serotypes persist as major causes of non-invasive pneumococcal pneumonia in Portugal despite declines in serotypes 3 and 19A (2012-2015)
Source: PLoS One. 2018 Nov 2;13(11):e0206912. doi: 10.1371/journal.pone.0206912 (PMC6214563; doi:10.1371/journal.pone.0206912)
Supplement: S2 Table — (PDF) [file pone.0206912.s004.pdf]

S2 Table. Serotype distribution of the isolates causing non-invasive pneumococcal pneumonia and invasive pneumococcal disease in adults in Portugal (2012–2014).

Serotype	n (%)[a]		OR[b]	$CI_{95\%}$	p value[c]
	NIPP (n=998)	IPD (n=1163)			
3	143 (14.3)	161 (13.8)	1.1	(0.8-1.3)	0.757
11A	80 (8.0)	49 (4.2)	2.0	(1.4-2.9)	**<0.001**
19F	59 (5.9)	27 (2.3)	2.6	(1.6-4.4)	**<0.001**
23A	50 (5.0)	26 (2.2)	2.3	(1.4-3.9)	**0.001**
19A	44 (4.4)	84 (7.2)	0.6	(0.4-0.9)	**0.006**
6C	42 (4.2)	28 (2.4)	1.8	(1.1-3.0)	0.020
9N	40 (4.0)	39 (3.4)	1.2	(0.7-1.9)	0.424
23B	38 (3.8)	12 (1.0)	3.8	(1.9-8.0)	**<0.001**
31	36 (3.6)	11 (0.9)	3.9	(1.9-8.6)	**<0.001**
NT	33 (3.3)	10 (0.9)	3.9	(1.9-9.0)	**<0.001**
22F	28 (2.8)	79 (6.8)	0.4	(0.2-0.6)	**<0.001**
15A	27 (2.7)	27 (2.3)	1.2	(0.7-2.1)	0.583
14	24 (2.4)	73 (6.3)	0.4	(0.2-0.6)	**<0.001**
15B/C	24 (2.4)	22 (1.9)	1.3	(0.7-2.4)	0.456
29/35B	24 (2.4)	26 (2.1)	1.1	(0.6-2.0)	0.886
8	23 (2.3)	123 (10.6)	0.2	(0.1-0.3)	**<0.001**
10A	22 (2.2)	18 (1.5)	1.4	(0.7-2.9)	0.267
16F	20 (2.0)	23 (2.0)	1.0	(0.5-1.9)	1
17F	20 (2.0)	9 (0.8)	2.6	(1.1-6.6)	**0.015**
6A	18 (1.8)	7 (0.6)	3.0	(1.2-8.6)	**0.014**
20	16 (1.6)	39 (3.4)	0.5	(0.2-0.9)	**0.009**
21	16 (1.6)	0 (0)	Inf	(4.5-inf)	**<0.001**
34	16 (1.6)	8 (0.7)	2.4	(0.9-6.4)	0.062
6B	15 (1.5)	15 (1.3)	1.2	(0.5-2.6)	0.715
35F	14 (1.4)	13 (1.1)	1.3	(0.5-2.9)	0.566
7F	13 (1.3)	61 (5.2)	0.2	(0.1-0.4)	**<0.001**
24F	12 (1.2)	23 (2.0)	0.6	(0.3-1.3)	0.173
33A	12 (1.2)	9 (0.8)	1.6	(0.6-4.2)	0.381
7C	10 (1.0)	6 (0.5)	2.0	(0.6-6.6)	0.215
23F	10 (1.0)	13 (1.1)	0.9	(0.3-2.2)	0.836
25A/38	9 (0.9)	8 (0.7)	1.3	(0.4-3.9)	0.631
35A	9 (0.9)	2 (0.2)	5.3	(1.1-50.3)	0.029
13	6 (0.6)	2 (0.2)	3.5	(0.6-35.6)	0.155
18C	6 (0.6)	7 (0.6)	1.0	(0.3-3.5)	1
37	6 (0.6)	0 (0)	Inf	(1.4-inf)	**0.010**
9V	5 (0.5)	9 (0.8)	0.6	(0.2-2.2)	0.593
4	4 (0.4)	23 (2.0)	0.2	(0.1-0.6)	**0.001**
12B	3 (0.3)	18 (1.5)	0.2	(0-0.7)	**0.003**

Serotype	n (%)[a]		OR[b]	$CI_{95\%}$	p value[c]
	NIPP (n=998)	IPD (n=1163)			
17A	3 (0.3)	0 (0)	Inf	(0.5-inf)	0.098
1	2 (0.2)	26 (2.2)	0.1	(0-0.4)	**<0.001**
10B	2 (0.2)	0 (0)	Inf	(0.2-inf)	0.213
10F	2 (0.2)	1 (0.1)	2.3	(0.1-137.7)	0.599
11B	2 (0.2)	2 (0.2)	1.2	(0.1-16.1)	1
18A	2 (0.2)	3 (0.3)	0.8	(0.1-6.8)	1
33F	2 (0.2)	3 (0.3)	0.8	(0.1-6.8)	1
47F	2 (0.2)	1 (0.1)	2.3	(0.1-137.7)	0.599
28A	1 (0.1)	1 (0.1)	1.2	(0-91.5)	1
35C	1 (0.1)	0 (0)	Inf	(0-inf)	0.462
36	1 (0.1)	0 (0)	Inf	(0-inf)	0.462
42	1 (0.1)	0 (0)	Inf	(0-inf)	0.462
5	0 (0)	1 (0.1)	0	(0-45.4)	1
6D	0 (0)	1 (0.1)	0	(0-45.4)	1
12A	0 (0)	2 (0.2)	0	(0-6.2)	0.503
16A	0 (0)	2 (0.2)	0	(0-6.2)	0.503
18F	0 (0)	2 (0.2)	0	(0-6.2)	0.503
19B	0 (0)	1 (0.1)	0	(0-45.4)	1
22A	0 (0)	1 (0.1)	0	(0-45.4)	1
24A	0 (0)	2 (0.2)	0	(0-6.2)	0.502
24B	0 (0)	1 (0.1)	0	(0-45.4)	1
33B	0 (0)	2 (0.2)	0	(0-6.2)	0.503
39	0 (0)	1 (0.1)	0	(0-45.4)	1

[a]IPD - invasive pneumococcal disease. NIPP – non-invasive pneumococcal pneumonia. Data from IPD were published previously [6].

[b]Odds ratios and 95% confidence intervals ($CI_{95\%}$) were used to measure the association between serotype and disease presentation.

[c]In bold are significant p-values ($p<0.05$) after FDR correction.
